# Chemometric Analysis of the Volatile Compounds Generated by *Aspergillus carbonarius* Strains Isolated from Grapes and Dried Vine Fruits

**DOI:** 10.3390/toxins10020071

**Published:** 2018-02-06

**Authors:** Zhan Cheng, Menghua Li, Philip J. Marriott, Xiaoxu Zhang, Shiping Wang, Jiangui Li, Liyan Ma

**Affiliations:** 1 College of Food Science and Nutritional Engineering, China Agricultural University, Beijing 10083, China; cz421420@cau.edu.cn (Z.C.); limenghua127@126.com (M.L.); zxxjoypeace@foxmail.com (X.Z.); wang744447@126.com (S.W.); 2 Australian Centre for Research on Separation Science, School of Chemistry, Monash University, Clayton, VIC 3800, Australia; philip.marriott@monash.edu; 3 Institute of Forestry, Xinjiang Agricultural University, Urumqi 830052, China; lijiangui1971@163.com; 4 Supervision, Inspection & Testing Center for Agricultural Products Quality, Ministry of Agriculture, Beijing 100083, China; 5 Key Laboratory of Safety Assessment of Genetically Modified Organism (Food Safety), Ministry of Agriculture, Beijing 10083, China

**Keywords:** ochratoxin A, *Aspergillus carbonarius*, untargeted profiling, chemometrics, biosynthetic pathway

## Abstract

Ochratoxin A (OTA) contamination in grape production is an important problem worldwide. Microbial volatile organic compounds (MVOCs) have been demonstrated as useful tools to identify different toxigenic strains. In this study, *Aspergillus carbonarius* strains were classified into two groups, moderate toxigenic strains (MT) and high toxigenic strains (HT), according to OTA-forming ability. The MVOCs were analyzed by GC-MS and the data processing was based on untargeted profiling using XCMS Online software. Orthogonal projection to latent structures discriminant analysis (OPLS-DA) was performed using extract ion chromatogram GC-MS datasets. For contrast, quantitative analysis was also performed. Results demonstrated that the performance of the OPLS-DA model of untargeted profiling was better than the quantitative method. Potential markers were successfully discovered by variable importance on projection (VIP) and *t*-test. (*E*)-2-octen-1-ol, octanal, 1-octen-3-one, styrene, limonene, methyl-2-phenylacetate and 3 unknown compounds were selected as potential markers for the MT group. Cuparene, (*Z*)-thujopsene, methyl octanoate and 1 unknown compound were identified as potential markers for the HT groups. Finally, the selected markers were used to construct a supported vector machine classification (SVM-C) model to check classification ability. The models showed good performance with the accuracy of cross-validation and test prediction of 87.93% and 92.00%, respectively.

## 1. Introduction

Ochratoxin A is a mycotoxin reported to be a potential human carcinogen (group 2B) defined by the International Agency for Research on Cancer (IARC) and it is common in grape and grape-related products [1,2]. *A. carbonarius* in section *Nigri* is known to infect grape and is a main source of ochratoxin A in grape products [3,4]. Identifying different toxigenic strains is a crucial task to control the safety of foodstuffs. A typical approach is to assess volatile organic compounds generated by the fungi [5,6,7].

Microbial volatile organic compounds (MVOCs) are generated by the metabolism of microorganisms such as bacteria and filamentous fungi [8,9,10,11]. The MVOCs have been studied for various reasons including predicting spoilage processes caused by microorganisms during the period of storing foodstuffs, taxonomy research to identify different fungal species [8,9] and investigating the relation between volatile compounds in indoor air environments with contamination by fungi [10,11]. Also, MVOCs have been used to discover the relationships with the mycotoxins [5,6,12,13]. For example, Jeleń et al. [13], investigated the volatile sesquiterpenes generated by both toxigenic and nontoxigenic *Fusarium sambucinum* strains, and the toxigenic strains produced more sesquiterpenes with greater chemical diversity compared with nontoxigenic strains. However, a later study investigated volatile compounds produced by *Aspergillus* strains with different OTA-forming ability and showed that the profile of volatiles generated by toxic strains could not be distinguished from non-toxic strains [5]. Therefore, further research needs to be applied to characterize different toxigenic strains. Selected previous studies were performed by gas chromatography-mass spectrometry (GC-MS) technology to analyze MVOCs based on relative quantitative data [5,6,12,13]. Limitations of this time-consuming quantitative analysis approach included incomplete peak resolution [14] and limited breadth of analysis [15].

Data analysis strategies have developed over many years and suggested chemometrics as a useful and efficient way to analyze large data sets generated by modern information-rich analytical techniques [15,16,17,18,19,20]. The data generated from GC-MS experiments exhibit high dimensionality with numerous variables, and in order to better understand the information between the different samples, untargeted metabolic fingerprinting of GC-MS data coupled with chemometrics has proven to be a robust tool [21,22]. However, untargeted profiling of MVOCs to distinguish different toxigenic strains is not always available or precisely identified in reference library data.

A critical step for metabolomics study is to analyze high-dimensional data generated from the GC-MS data. A variety of chemometrics methods have been developed to project the multi-dimensional data to lower dimensions and explore the differences between group samples [23] including partial least squares discriminant analysis (PLS-DA) [24], orthogonal projection to latent structures discriminant analysis (OPLS-DA) [25], principle component analysis (PCA) and support vector machine (SVM) [26,27]. Of these, PLS-DA is one of the most attractive classification methods in chemometrics and has been successfully implemented in metabolomics research [22,28,29]. OPLS-DA is an extension of PLS-DA, which improves the interpretation of constructed models by removing variance orthogonal to the variation of interest [30]. The advantage of OPLS-DA is that one single component is used to predict the group or class whereas the rest of the components are used to define the variation orthogonal to the first predicting component [31,32]. In addition, PLS-DA and OPLS-DA can provide statistical information, such as loading weight, sensitivity ratios (SR), regression coefficients and variable importance on projection (VIP), which can be performed to find out important variables [28,33,34]. Among these, VIP is popular in metabolomics in order to choose potential markers or discriminate metabolites [15]. SVM is a so-called machine-learning strategy and it is a powerful modeling tool to solve classification problems [35,36]. The advantage of this method is its flexibility to solve both linear and non-linear problems [23].

Until now, it is very difficult to distinguish different OTA contamination levels in grape and grape production using volatile compounds, due to the fact that grape products have very complex volatile composition, which will likely interfere with the MVOCs related specifically to OTA generation. Therefore, it is necessary to clearly understand the MVOCs generated from different toxigenic *A. carbonarius* and ideally identify relevant biomarkers specific to the presence of OTA. Our previous work has demonstrated the capacity to predict the OTA content using volatile compounds with PLS regression methods [37]. However, due to the shortage of negative *A. carbonarius* strains, namely non-toxigenic or moderate toxigenic strains, the character of moderate toxigenic (MT) and high toxigenic (HT) strains could not be applied to chemometrics analysis. In this study, as model fungi strains, two moderate toxigenic strains were selected. An untargeted metabolic profiling approach was carried out to explore the volatile information generated by GC-MS for selected *A. carbonarius* strains. In order to validate its feasibility, traditional quantitative analysis was also performed. The chemometrics techniques were used as robust tools for extracting the volatile character of different toxigenic strains. In this study, the potential for MVOCs with chemometrics to be used to recognize different toxigenic strains was comprehensively investigated. Subsequently, exploring potential biomarkers to provide clues for metabolism pathways may be suggested.

## 2. Results and Discussion

### 2.1. Toxigenic Investigation of A. carbonarius Strains

**Figure 1:**
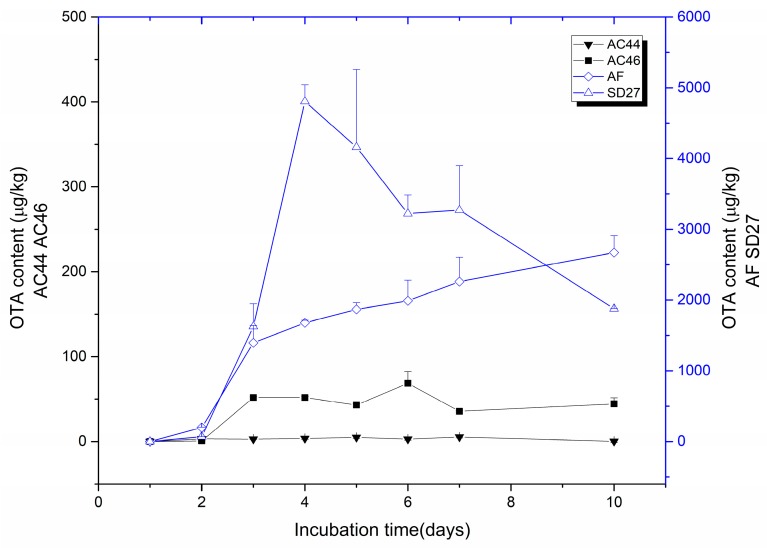
OTA content of the strains incubated on CYA culture medium.

The OTA producing ability of four strains (AC44, AC46, SD27 and AF) during incubation periods in Czapek Yeast Extract Agar (CYA) culture medium were analyzed. On the basis of the experiment the strains could be divided into two classes, namely MT strains (AC44 and AC46) and HT strains (SD27 and AF). The amount of OTA produced by the investigated strains is shown in Figure 1. The content of OTA varied especially according to different HT and MT groups. For SD27 strains, the OTA synthesis commenced from the 2nd day, then sharply increased to the highest content (4808 μg/kg) at the 4th day, then decreased by about 2.5 fold over the following days. The other HT strain AF showed a different trend compared with SD27 strain, with the content of OTA gradually rising over the 10-day measurement period to 2670 μg/kg. Regarding the MT strains, AC44 and AC46 showed a similar trend that the OTA synthesized from the 2nd day remained stable over the remaining days. The content of OTA was 0–5.4 and 0.8–68.6 μg/kg for AC44 and AC46 strains, respectively, being some 2000–5000 of μg/kg less than the HT group.

### 2.2. GC-MS Profiles of Different Toxigenic Strains

**Figure 2:**
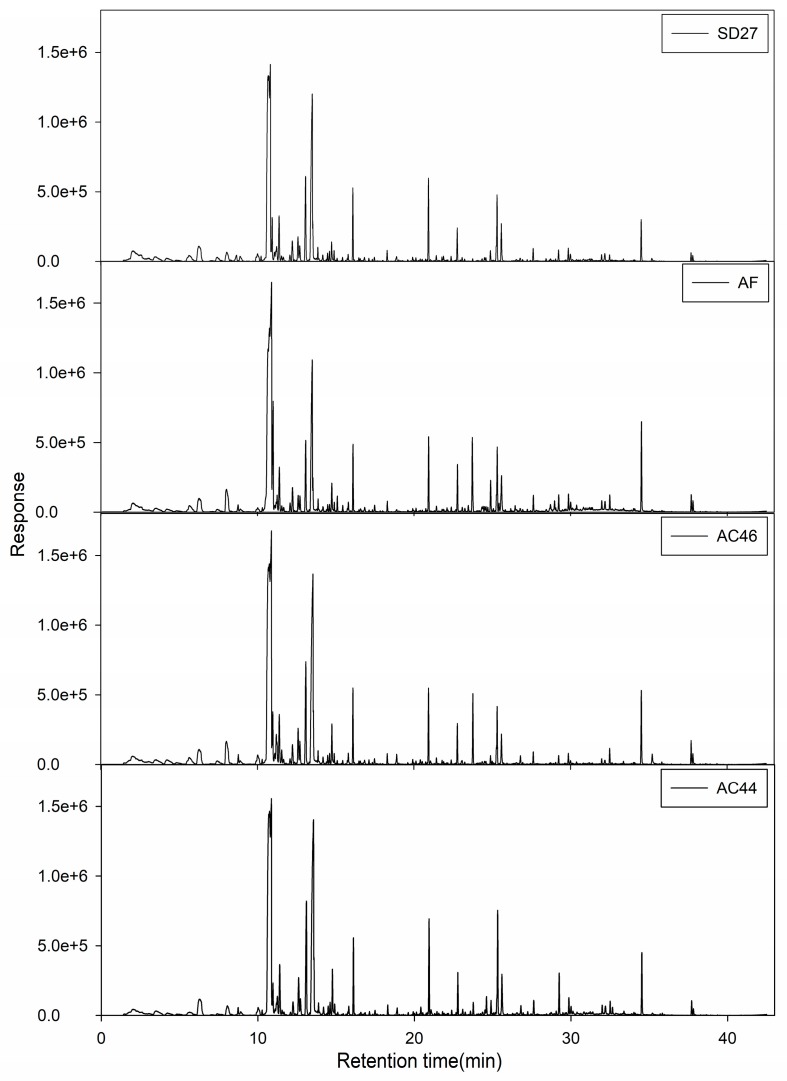
The typical total ion chromatograms (TICs) of the two groups of strain.

The total ion chromatograms (TICs) of MVOCs profiling for different toxigenic strains grown at the 3rd day are shown in Figure 2 and the resulting data are shown in Table 1. In totally, fifty-two MVOCs were qualitatively and quantitatively analyzed in detail. Among these, nineteen MVOCs were unambiguously identified using the authorized chemical standards. The rest are tentatively reported by comparing the MS profile and retention indices (RIs) with literature values in the NIST 11 database. These MVOCs included 3 alcohols, 5 aldehydes, 3 ketones, 9 esters, 12 sesquiterpenes, 18 hydrocarbons and two other compounds. 

1-Octen-3-ol and other compounds with eight carbons, (*E*)-2-octen-1-ol, 1-octanol, octanal, (*E*)-2-octenal, 1-octen-3-one and 3-octanone were both found in both MT and HT strains. These 8-carbon compounds may be synthesized by oxidation of linoleic acids [38] and were isolated from numerous molds, such as *A. ochraceus*, *A. oryzae* and *A. niger* [39,40]. They could be recognized as indicators for the invasion of molds, especially when 1-octen-3-ol was detected, which contributed to a mushroom flavor [38]. 

The esters generated by the four strains include 7 fatty acid methyl esters and two other esters, methyl benzoate and methyl-2-phenylacetate. The fatty acid methyl esters may derive from enzyme catalyzed reactions between alcohols and acyl-CoA [41]. Methyl-2-phenylacetate is an important flavor compound in wine, which contributes to the fruity notes of wine aroma [42] and it was first detected in *A. carbonarius* incubated in CYA medium.

Considering the hydrocarbons, 18 compounds were identified, including styrene, 17 alkanes and isoalkanes, of which the carbon backbone ranged from C11 to C18. Styrene is an 8-carbon compound and is derived from phenylalanine by the shikimic acid pathway [43,44]. It has been found in some species of *Penicillium* and could be a potential indicator of food spoilage, capable of producing off-flavors [45]. Alkanes and isoalkanes were found in *A. carbonarius* and their diversity was mainly determined elsewhere by the different carbon source used in the culture medium [46].

There were 12 terpenoids found in four strains, including limonene, *p*-cymene and 10 sesquiterpenes. Limonene is a commonly identified metabolite generated by *P. glabrum*, *P. roqueforti*, *A. flavus*, and *A. ochraceus* [5,47] and it was found in all the strains. Sesquiterpenes are regarded as representative compounds showing different characters with different toxigenic strains, such as *Fusarium sambucinum* [13], *A. flavus* [7] and *P. roqueforti* [12]. In our study, 10 sesquiterpenes were detected and in particular, β-cedrene, β-chamigrene, β-himachalene and cuparene were only detected in AF strains. By contrast, β-farnesene was absent in AF strains.

Of the other compounds, 3-furanacetic acid, 4-hexyl-2,5-dihydro-2,5-dioxo- was found in all strains and it was first detected in *A. carbonarius* in our previous work [37]. The content of this compound reached a maximum at the 2nd day, and sharply declined from the 3rd to the 10th day. This compound may not be regarded as a specific compound for different toxigenic strains because it showed the same trend in both MT and HT strains.

In summary, the volatile profile of these two groups were similar except the AF strain, which has a unique sesquiterpenes pattern. The differences between them were confusing and the procedure of qualitative analysis and quantitative analysis is complicated and time-consuming. Therefore, further analysis is necessary to explore the useful information which can be used to distinguish them reliably.

### 2.3. Chemometrics for Analyzing the Differences of Two Group Strains

The MVOCs data obtained by GC-MS were submitted to XCMS online to generate the adjusted EIC automatically. In total, 829 EICs were obtained and all the EICs were normalized by the internal standard ion fragment which was coded as M57T23 using the ion mass *m*/*z* 57. Then, an 828 × 84 dataset was used for the subsequent chemometrics analysis. 

**Figure 3:**
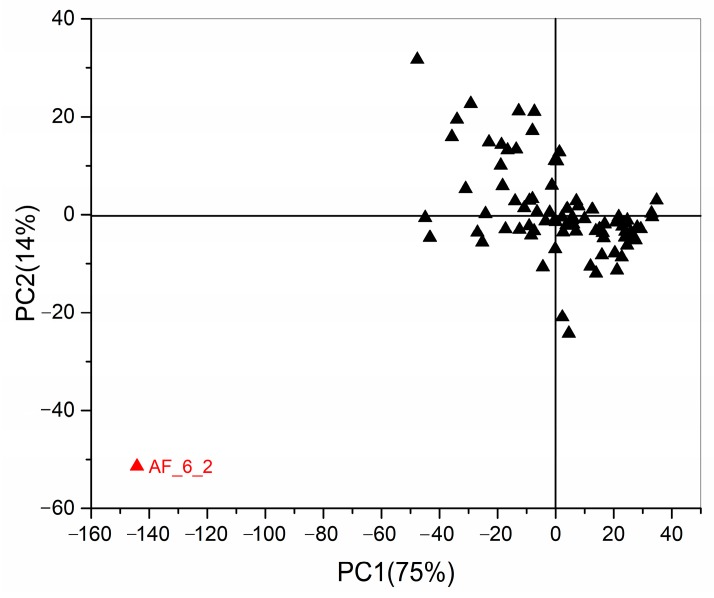
PCA score plot for the metabolic profile of four strains.

In order to find outliers, an unsupervised pattern recognition method (PCA) was performed in this study. All data were scaled using a Pareto scaling method. As shown in Figure 3, PC1 accounted for 75% and PC2 accounted for 14% of total variation. An outlier (coded as AF_6_2 in red) stood out from the major group of samples. It was caused by the variation of the internal standard, which meant that the content of the internal standard was significantly lower in the sample marked as AF_6_2 than others. This sample was excluded from further analysis.

**Figure 4:**
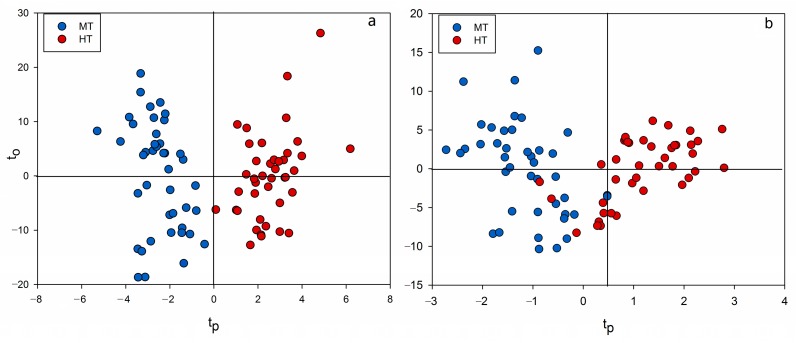
OPLS-DA score plots of two models, (**a**) untargeted profiling; (**b**) quantitative analysis.

After that, two OPLS-DA models were carried out to differentiate between MT and HT groups. For untargeted profiling method, the result is shown in Figure 4a, the OPLS-DA model for CYA medium demonstrated that the fungi were clearly divided into two clusters according to their different toxigenic ability. The model generated one predictive and four orthogonal (1 + 4) components with R^2^ of 85.0% and Q^2^ was 67.4%. In order to prove the robustness of this untargeted profiling method, the data obtained from quantitative analysis of GC-MS was also performed as a control method. Another OPLS-DA model based on quantitative analysis (the dataset was 52 × 83) was constructed and the result is shown in Figure 4b. Some overlapping occurred in the two-dimension score plot. Besides, the model generated one predictive and five orthogonal (1 + 5) components with R^2^ and Q^2^ values of 68.4% and 50.9%, respectively, which means that the performance of this model was not as good as the OPLS-DA model based on the untargeted GC-MS profiling.

### 2.4. Discovery of Potential Markers of HT and MT Strains

**Figure 5:**
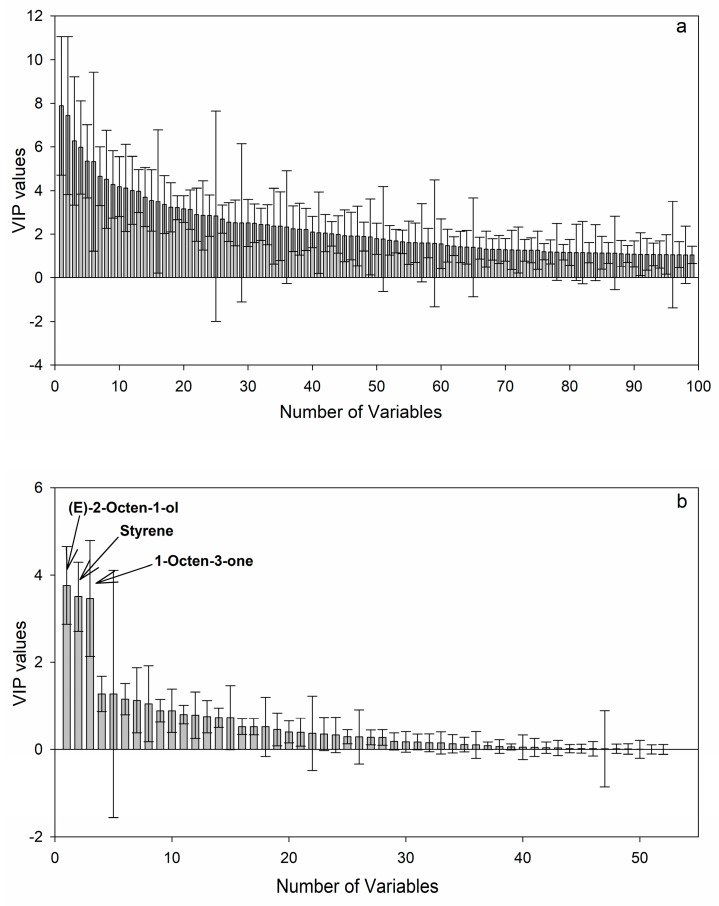
Variable importance on projection (VIP) plot scores for two models, (**a**) untargeted profiling; (**b**) quantitative analysis.

The potential markers discovery is a critical step for metabolomics studies [28]. The process of selecting informative metabolites was important for finding the differences between HT and MT strains and it could provide clues of their different metabolism pathways. Potential markers were then selected using VIP values based on the untargeted profiling method. The plot of VIP value (first 100 variables) with standard error is shown in Figure 5a. The potential markers were selected based on VIP value higher than 1.5 [21,22] and *p* < 0.05 according to the *t*-test. Besides, metabolites with error bars extending beyond zero, which showed no statistic meaning, were also excluded. Finally, 39 extracted ion variables were obtained and these variables were identified using ion information and retention times. In total, 12 compounds were identified and the relative content (normalized by the internal standard ion fragment) is shown in Table 2.

These volatile compounds included, 1 alcohol, 1 aldehyde, 1 ketone, 1 ester, 3 hydrocarbons, 2 sesquiterpenes and 4 unidentified compounds. Of these, (*E*)-2-octen-1-ol, octanal, 1-octen-3-one, styrene, limonene and 3 unidentified compounds (*m*/*z* was 91, 91 and 165) were selected as the important metabolites for AC44 and AC46 strains. The abundance of these compounds was significantly higher than those generated by high toxigenic strains.

**Figure 6:**
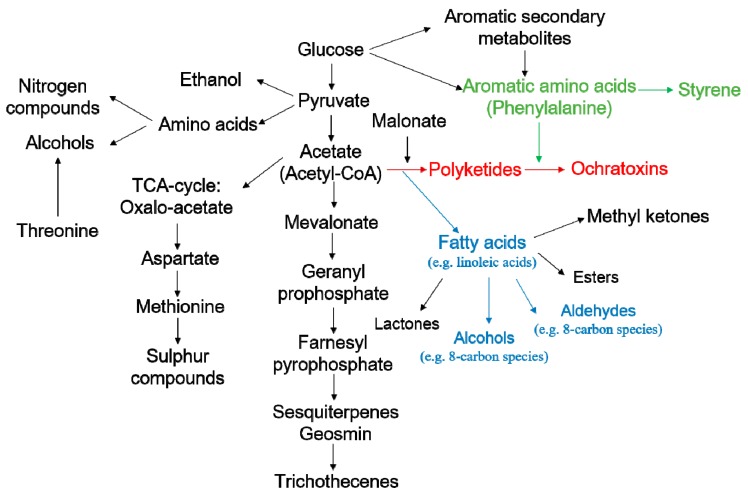
The pathways of MVOCs involved in the production of different secondary metabolites.

The result was similar to previous studies, that the non-toxigenic strains synthesized more volatile compounds than the toxigenic strains [5]. The reason for abundant C8-compounds, (*E*)-2-octen-1-ol, octanal and 1-octen-3-one, in MT strains may be explained by the metabolic pathway leading to the formation of MVOCs and OTA, which provides important clues to the relationship between mycotoxin formation and various groups of volatiles (Figure 6) [41]. The polyketide skeleton formation (marked in red) is a critical step of OTA biosynthesis, which requires acetate and malonate with the activity of polyketide synthases [48]. Meanwhile, the fatty acid formation pathway (marked in blue) is also derived from acetate and malonate via the acetate-malonate pathway, which forms a competitive relationship with polyketide skeleton formation [41]. According to that, we speculate that less OTA biosynthesis may lead to more fatty acid formation. As a result, more eight carbon compounds, octanal, (*E*)-2-octen-1-ol and 1-octen-3-one, are synthesized from fatty acid [38]. In particular, 1-octen-3-one was a possible precursor of 1-octen-3-ol being produced via reduction or autoxidation [49,50]. Regarding the hydrocarbons, styrene was identified as the important metabolite for the MT strains and the result was in agreement with a previous study [51]. From the pathway marked in green (Figure 6), it can be assumed that less phenylalanine was used to produce the ochratoxins, and the surplus was used to synthesize more styrene than the HT strains. Limonene was firstly selected as a potential marker for MT strains, though the reason for this is not clear and needs to further research.

For HT strains, 2 identified sesquiterpenes, namely cuparene and (*Z*)-thujopsene, and 1 ester, methyl octanoate, were selected as potential markers. There is an unknown compound identified as a potential marker for HT strains, which has ion information of *m*/*z* 69, 84, 55. The sesquiterpenes have been considered as a main difference between different toxigenic strains, such as *Aspergillus flavus* [7]. Results from previous study showed the *Aspergillus* strains which could synthesize OTA produced more sesquiterpenes [5]. These two sesquiterpenes were firstly identified as potential markers for high toxigenic *A. carbonarius* strains. As for methyl octanoate, it has been showed that it may be play an important role in the OTA biosynthesis [37].

For comparison, VIP values were also calculated based on quantitative analysis and similar but not integrated results were obtained that three metabolites including 1-octen-3-one, 2-octen-1-ol and styrene (VIP value beyond 1.5) were selected as potential markers (Figure 5b). This result showed the robustness of untargeted profiling for analyzing the MVOCs to discover differences between HT and MT strains.

### 2.5. SVM-C Pattern Recognition Based on Potential Markers

**Figure 7:**
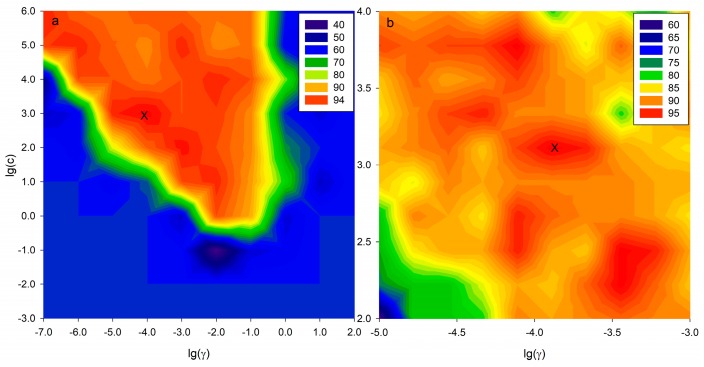
Grid search for optimizing parameters (C, γ). (**a**) Coarse search and (**b**) finder search. The optimal parameters selected by grid search are marked as “×”.

To check the classification ability of the selected variables, namely, the potential markers for different group strains explored by the untargeted profile method, the SVM-C model was built by using these fragmentations. The dataset was 39 × 83 and the RBF was applied as kernel function of the SVM-C model in our study. Optimizing the appropriate SVM-C parameters (C, γ) is an important procedure to provide good prediction performance. In addition, a 10 × 10 coarse grid search was performed to adjust for the proper parameters. 3-fold cross validation was used to check the performance of SVC models. The result is shown in Figure 7 and the optimal pair of parameters according to the coarse search was marked with “×” and it was (10^3^, 10^−4^) (Figure 7a). Next, a finder grid search on the neighbor of (10^3^, 10^−4^) was conducted and (1.29 × 10^3^, 1.29 × 10^−4^) was selected as optimal parameters (Figure 7b). When the best parameter (C, γ) was found, the training set was trained again to generate the classifier.

Finally, the test set was classified using the SVM-C model. The classification result is shown in Table 3 and the accuracy of cross-validation and test prediction was 87.93% and 92.00%, respectively. The same procedure was performed using the full 828 × 83 dataset and accuracy of cross-validation and test prediction was 77.59% and 84.00%, respectively. These results showed the robustness of the SVM-C model using the potential markers selected by the untargeted profiling approach.

## 3. Conclusions

In the present study, the untargeted profile of MVOCs based on GC-MS data was firstly introduced coupled with chemometrics analysis to distinguish different toxigenic *A. carbonius* strains. Comparing with traditional quantitative analysis, the untargeted profile method has the potential to provide comprehensive information and enhance the model performance. Furthermore, the identified potential markers, selected by VIP values and *t*-test, could be used for classifying HT strains from MT strains and they may provide clues of metabolite pathway of different toxigenic strains. We reiterate that this study is preliminary, and the ability to distinguish different levels of OTA contamination in grape and grape products with this novel system approach need to be further tested on more grape and grape-product samples.

## 4. Materials and Methods

### 4.1. Chemicals

Volatile standards (Table 1), C8-C40 n-alkane series and ochratoxin A (OTA) standard were purchased from Sigma Aldrich (St. Louis, MO, USA). Highly purified water was obtained from a Milli-Q Gradient system (18 kΩ, Millipore, Bedford, MA, USA). Glacial acetic acid, acetonitrile and formic acid (99% purity) were HPLC grade and were obtained from Merck (Darmstadt, Germany).

### 4.2. Fungi and Cultivation

Four *A. carbonarius* strains separated into two groups were used in this study, namely HT and MT groups. The HT strains, including CCTCC AF2011004 (coded: AF) and AF 2015027 (coded: SD27) strains, were isolated from grapes and dried vine fruits, respectively [37]. The MT strains, including AC44 and AC46 strains, were isolated from grapes [52] and kindly provided by Dr. P. I. Natskoulis (Department of Food Science and Human Nutrition, Agricultural University of Athens, Greece). Strain spores used for spore suspension were incubated on Malt Extract Agar (AOBOX, Beijing, China) culture medium at 25 °C for 7 days. Afterwards, the spores were diluted with an aqueous solution including 0.05% Tween 80 (*v/v*) to prepare strain spore suspension (concentration was10^5^ spores/mL).

For fungi cultivation, Czapek Yeast Extract Agar (CYA; AOBOX, Beijing, China) culture medium (10 mL) was added to a 30 mL head space vial. Then, the vial was autoclaved for 20 min at 121 °C and the spore suspension (100 μL) was added to each vial and capped with cotton plugs. Afterwards, the strain was incubated at 25 °C in the dark under stationary conditions from 2nd to 7th and 10th days. The same volume of the autoclaved medium with 100 μL of 0.05% Tween 80 aqueous solution was used as control samples. All the experiments were performed in triplicate and a total of 84 samples (4 strains incubated over a seven-day period and performed in triplicate) were prepared for GC-MS analysis.

### 4.3. GC-MS Analysis

The GC-MS analyses followed our previous work [37]. In brief, tetradecane was dissolved in methanol and the solution was used as internal standard. Before extraction, 10 μL of tetradecane (5.0 mg/L) were placed into the bottom of the vial. The sample vial caps were replaced by crimp-top silicon rubber caps with a Teflon layer and maintained at 60 °C in a water bath. Subsequently, the volatile compounds were extracted by SPME with a 2 cm, 50/30 µm, coated DVB/CAR/PDMS fiber supplied by Supelco (Bellefonte, PA, USA) and the extraction time was 60 min. 

The determination was conducted using an Agilent 7890 gas chromatograph (Agilent, Santa Clara, CA, USA) fitted with an Agilent 5975C mass spectrometer (Agilent). Volatile compounds were injected in the splitless mode injector (splitless time of 0.75 min) heated at 240 °C for 7 min and separated on a DB-5 capillary column (30 m × 0.25 mm × 0.25 μm; Agilent). Helium was used as carrier gas with a constant flow rate at 1.0 mL/min. The temperature program was as follows: 35 °C for 1 min, and then increased to 230 °C at 5 °C /min, and finally increased to 280 °C at 20 °C /min. Electron ionization (EI-MS) mode was carried out at 70 eV and a mass scan range from *m*/*z* 35 to 330 atomic mass units (amu).

### 4.4. Ochratoxin A Analysis

The OTA analysis followed our previous work [53,54]. The ultrasound-assisted extraction was used to extract OTA from culture sample with 10 mL of methanol aqueous solution (7:3, *v*/*v*) for 30 min. This procedure was repeated twice with 5 mL of solution each time. Extracts were filtered through a Whatman glass microfiber filter (Sigma Aldrich) to remove the hyphae and spores. Subsequently, the resultant extract was filtered through 0.22 μm nylon syringe filters (Lanyi, Beijing, China) before high-performance liquid chromatography (HPLC) analysis. The liquid chromatography (LC) system consisted of a fluorescence detector (RF-20 Axs) and a pump (LC-20 AT) (Shimadzu Scientific Instruments, Kyoto, Japan) with a 5 μm Prodigy ODS3, 100 A, 250 × 4.6 mm analytical column (Phenomenex, Torrance, CA, USA). Separation was carried out by using isocratic elution with isometric mobile phase A (composed by a water and glacial acetic acid (99:1, *v*/*v*) solution) and mobile phase B (composed of acetonitrile and glacial acetic acid (99: 1, *v*/*v*) solution), at a rate of 1.0 mL/min and 20 μL injection. Detection of OTA was performed using 333 nm and 460 nm as wavelength settings for excitation and emission, respectively. Quantification of OTA was carried out by measuring its peak area according to a five-point calibration curve between 3.2 and 4000 μg/L, which was constructed by five serial dilutions of the OTA standard solution. The squared correlation coefficient (r^2^) was 1. 

### 4.5. Data Processing

Untargeted metabolic profiling analysis was performed for the fungi volatile compounds. Raw data were processed with multiple procedures, containing filtering, feature detection, alignment and normalization, according to the pipeline described by Katajamaa and Orešič [55]. For this purpose, the freely available software XCMS online (http://xcmsonline.scripps.edu) was introduced in our study [56]. Raw data were transferred to NetCDF files using the MSD ChemStation software (Agilent). Afterwards, data were extracted using the centWave algorithm, which collects regions including potentially useful mass information in the chromatographic data and applies continuous wavelet transformation (CWT) [15]. The advantage of this method is detection of both strong and weak peak responses while maintaining a high sensitivity and low false discovery rate (FDR) [57]. The XCMS online parameters were optimized to extract the maximum information possible according to the protocol described by González-Domínguez et al. [21], According to the character of our data, the setting was S/N threshold 3 and minimum peak width was 3 s. The remaining parameters were set as default. Pre-processed data were then exported as .csv files for further analysis using chemometrics.

The processing pipeline of quantitative analysis comprised the following steps: deconvolution, library-based identification, and alignment [58]. Identification and deconvolution comprise the main procedures of data processing, while alignment is a validation procedure for identification. For deconvolution, the open source software, automated mass spectra deconvolution (AMDIS) was used to process the GC-MS data. Next, alignment was performed relying on retention index (RI) similarity. RI data were calculated automatically by AMDIS software, with the help of performing a series of n-alkanes (C7-C40) under the same chromatographic conditions. Subsequently, MVOCs were determined according to RIs of available standards and obtained mass spectra compared with corresponding volatile standards in the NIST11 MS database. Considering those volatile compounds without reference standards, tentative identifications were conducted based on comparison of mass spectra with those of the NIST11 MS database with match quality higher than 700 [59] and RIs found in literature. For quantification, a specific ion was extracted for each volatile compound (Table 1), which was generally the most abundant. The respective area of the specific ion was then calculated. Afterwards, relative areas of volatile compounds were obtained compared to that of the *m*/*z* 57 ion of the internal standard (tetradecane).

### 4.6. Chemometrics Analysis

Identified volatile compounds and extract ion chromatogram (EIC) data generated by XCMS were both subjected to chemometrics analysis by OPLS-DA to compare MVOCs profiles, by means of SIMCA-P™ software (Version 13.0, UMetrics AB, Umeå, Sweden). Before constructing the OPLS-DA model, data were normalized using a Pareto scaling strategy to reduce the impact of artifacts and noise in the models, which is positive for the model’s predictive ability [60]. For evaluation of the model performance, two parameters were calculated, namely the R^2^ representing total explained variance and cumulative Q^2^ that represents the fraction of the variation of Y which can be predicted by the cross validation model [30]. Potential biomarkers were chosen from VIP generated from the OPLS-DA model. This variable selection method was described by Chong and Jun [61]. The higher the absolute value of VIP, the more important the corresponding variable [26]. Furthermore, potential markers identified by VIP were screened out by *t*-test (*p*-values below 0.05). 

### 4.7. Support Vector Machine Classification

Support vector machine (SVM) is a machine-learning strategy, which was originally introduced by Vapnik and co-workers [26,27]. In recent years, it has been widely used in different research due to its ability in prediction for both classification (SVM) [35,36] and regression [62,63]. When used for classification, the basic idea of the support vector classification is that a separated set of binary labeled training data was given with a hyper-plane which maximizes the distance from the two classes of patterns [64]. The advantage of this technique is its flexibility in the choice of the kernel function which allows the classification of two groups of samples, and this kernel can be used to select either linear or non-linear problems [23]. Besides, some of the extensively used kernel functions including linear, sigmoid, polynomial and radial basis function (RBF) can be carried out to construct models. Among these, the RBF is popular in many problems [65,66] and was chosen in our study. For RBF kernel function, two parameters are kernel width (γ) and regularization parameter (C), and the classification result of the given data are affected by the pairs of parameters. Therefore, parameter optimization is necessary before building the model [67]. In this study, the parameters of RBF were optimized by the grid search strategy using the n-fold cross validation approach. This method is conducted in two steps. Firstly, a coarse grid is applied with an exponentially growing sequence of (C, γ) (e.g., C = 10^−7^, …, 10^2^ and γ = 10^−3^, …, 10^6^). Secondly, a finder grid search on that region can be conducted to optimize the parameter (C, γ), which was used to perform the final training process. The SVM-C model consisted of both training and test datasets, which represented 70% (*n* = 58) and 30% (*n* = 25) of the data by random selection in the database. The SVM-C model was performed on The Unscrambler X 10.4 (CAMO Software, Oslo, Norway).

## Figures and Tables

**Table 1:** Volatile metabolites of four strains extracted from the CYA culture medium.

NO.	REF.RI ^1^	RI	Name	Identification Methods ^2^	Ion ^3^	CYA Culture Medium
			**Alcohols**			
1	979	980	1-Octen-3-ol	Std, MS, RI	57	AC44, AC46, AF, SD27
2	1069	1067	(*E*)-2-Octen-1-ol	Std, MS, RI	57	AC44, AC46, AF, SD27
3	1069	1070	1-Octanol	MS, RI	56	AC44, AC46, AF, SD27
			**Aldehydes**			
4	1001	1001	Octanal	MS, RI	43	AC44, AC46, AF, SD27
5	1057	1055	(*E*)-2-Octenal	Std, MS, RI	41	AC44, AC46, AF, SD27
6	1102	1103	Nonanal	MS, RI	57	AC44, AC46, AF, SD27
7	1115	1107	(*E*,*E*)-2,4-Octadienal	MS, RI	81	AC44, AC46, AF, SD27
8	1313	1314	(*E*,*E*)-2,4-Decadienal	MS, RI	81	AC44, AC46, AF, SD27
			**Ketones**			
9	978	976	1-Octen-3-one	MS, RI	55	AC44, AC46, AF, SD27
10	984	985	3-Octanone	Std, MS, RI	43	AC44, AC46, AF, SD27
11	1290	1291	2-Undecanone	MS, RI	43	AC44, AC46, AF, SD27
			**Esters**			
12	1092	1093	Methyl benzoate	MS, RI	105	AC44, AC46, AF, SD27
13	1120	1123	Methyl octanoate	MS, RI	74	AC44, AC46, AF, SD27
14	1255	1254	Methyl-2-phenylacetate	MS, RI	104	AC44, AC46, AF, SD27
15	1326	1322	Methyl decanoate	MS, RI	74	AC44, AC46, AF, SD27
16	1723	1723	Methyl tetradecanoate	MS, RI	74	AC44, AC46, AF, SD27
17	1823	1825	Methyl pentadecanoate	MS, RI	74	AC44, AC46, AF, SD27
18	1927	1926	Methyl hexadecanoate	Std, MS, RI	74	AC44, AC46, AF, SD27
19	2096	2095	Methyl linoleate	Std, MS, RI	67	AC44, AC46, AF, SD27
20	2100	2102	Methyl oleate	MS, RI	55	AC44, AC46, AF, SD27
			**Terpenoids**			
21	1024	1024	*p*-Cymene	MS, RI	119	AC44, AC46, AF, SD27
22	1028	1028	Limonene	MS, RI	68	AC44, AC46, AF, SD27
23	1412	1411	Longifolene	MS, RI	161	AC44, AC46, AF, SD27
24	1416	1417	α-Cedrene	Std, MS, RI	119	AC44, AC46, AF, SD27
25	1428	1426	β-Cedrene	MS, RI	161	AF
26	1435	1436	(*Z*)-Thujopsene	MS, RI	119	AC44, AC46, AF, SD27
27	1435	1438	α-Bergamotene	MS, RI	93	AC44, AC46, AF, SD27
28	1458	1457	β-Farnesene	Std, MS, RI	41	AC44, AC46, SD27
29	1481	1481	β-Chamigrene	Std, MS, RI	189	AF
30	1505	1505	β-Himachalene	MS, RI	119	AF
31	1509	1510	Cuparene	Std, MS, RI	132	AF
32	1563	1563	(*E*)-Nerolidol	Std, MS, RI	41	AC44, AC46, AF, SD27
			**Hydrocarbons**			
33	893	889	Styrene	Std, MS, RI	104	AC44, AC46, AF, SD27
34	1100	1100	Undecane	Std, MS, RI	57	AC44, AC46, AF, SD27
35	1200	1199	Dodecane	Std, MS, RI	57	AC44, AC46, AF, SD27
36	1300	1299	Tridecane	Std, MS, RI	57	AC44, AC46, AF, SD27
37	1318	1326	Decane, 2,3,5,8-tetramethyl-	MS, RI	57	AC44, AC46, AF, SD27
38	1400	1400	Tetradecane	Std, MS, RI	57	Internal standard
39	1460	1462	Tetradecane, 4-methyl-	MS, RI	43	AC44, AC46, AF, SD27
40	1500	1499	Pentadecane	Std, MS, RI	57	AC44, AC46, AF, SD27
41	1564	1562	Pentadecane, 2-methyl-	MS, RI	43	AC44, AC46, AF, SD27
42	1570	1569	Pentadecane, 3-methyl-	MS, RI	57	AC44, AC46, AF, SD27
43	1600	1600	Hexadecane	Std, MS, RI	57	AC44, AC46, AF, SD27
44	1649	1648	Pentadecane, 2,6,10-trimethyl-	MS, RI	57	AC44, AC46, AF, SD27
45	1666	1663	Hexadecane, 2-methyl-	MS, RI	57	AC44, AC46, AF, SD27
46	1700	1700	Heptadecane	Std, MS, RI	57	AC44, AC46, AF, SD27
47	1703	1706	Pristan	MS, RI	57	AC44, AC46, AF, SD27
48	1765	1763	Heptadecane, 2-methyl-	MS, RI	57	AC44, AC46, AF, SD27
49	1770	1771	Heptadecane, 3-methyl-	MS, RI	57	AC44, AC46, AF, SD27
50	1800	1800	Octadecane	Std, MS, RI	57	AC44, AC46, AF, SD27
51	1806	1810	Phytane	MS, RI	57	AC44, AC46, AF, SD27
			Others			
52	1181	1182	Naphthalene	MS, RI	128	AC44, AC46, AF, SD27
53	-	1484	3-Furanacetic acid, 4-hexyl-2,5-dihydro-2,5-dioxo-	MS	126	AC44, AC46, AF, SD27

^1^ REF.RI = literature retention index, obtained from the NIST11 database. The column type selected in NIST11 database for RI values is a DB-5 column (30 m × 0.25 mm × 0.25 μm). If not available, the RI values of HP-5 column (30 m × 0.25 mm × 0.25 μm) was chosen. ^2^ Identification Methods = Std (authentic standard retention time); MS (Mass spectrum) with minimum match of 70%; RI (Retention Index). ^3^ Ion = quantification ion response.

**Table 2:** Potential markers selected by VIP values and *t*-test.

NO.	Potential Markers	Retention Time/Min	Ion Information	Relative Content ^1^	
				MT	HT
1	Styrene	8.001–8.004	103, 78, 77, 104, 51, 105	0.13–29.77 *	0.08–13.21
2	1-Octen-3-one	10.627–10.672	97, 70, 111, 98, 83, 55	1.46–114.32 *	4.00–86.63
3	Octanal	11.378	55	0.03–2.95 *	0.04–1.43
4	Limonene	12.232	91	0.17–9.21 *	0.04–0.63
5	2-Octen-1-ol	13.408–13.466	68, 95, 58, 81, 54, 110, 82, 41, 39, 57, 55, 69, 67, 56	0.51–75.13 *	2.00–57.36
6	Methyl octanoate	15.091	74	0.03–0.14	0.03–0.56 *
7	Unknown	15.438–15.446	69, 84, 55	0.02–0.44	0.03–1.08 *
8	Unknown	20.402	91	0–0.82 *	0–0.25
9	Unknown	21.057	91	0–0.27 *	0–0.07
10	Thujopsene	23.718–23.756	204, 121, 105	0–0.67	0–4.1 *
11	Unknown	24.599	165	0.05–0.47 *	0.02–0.23
12	Cuparene	25.542	132	0–0.01	0–1.3 *

^1^ = Relative content (equivalent of tetradecane %) of all samples in each group. * Potential markers for each group strains are marked in bold type letter. This is according to criteria: significant value (*p* < 0.05) in statistical analysis (*t*-test) and variable important on projection (VIP) beyond 1.50.

**Table 3:** Performance of SVM-C model.

Variable Selection	Optimized Parameters	No. Variables	Data Sets	Accuracy (%)
Full variables	C = 4.64 × 10^2^	829	Cross-Validation	77.59
	γ = 1.67 × 10^−4^		Test	84.00
VIP method	C = 1.29 × 10^3^	39	Cross-Validation	87.93
	γ = 1.29 × 10^−4^		Test	92.00
